# Intratumoral ^18^F-FLT infusion in metabolic targeted radiotherapy

**DOI:** 10.1186/s13550-019-0496-7

**Published:** 2019-04-11

**Authors:** Thititip Tippayamontri, Brigitte Guérin, René Ouellet, Otman Sarrhini, Jacques Rousseau, Roger Lecomte, Benoit Paquette, Léon Sanche

**Affiliations:** 1 Department of Nuclear Medicine and Radiobiology, Faculty of Medicine and Health Sciences, Université de Sherbrooke, Sherbrooke, QC Canada; 2 Center of Radiotherapy Research, Faculty of Medicine and Health Sciences, Universite de Sherbrooke, Sherbrooke, QC Canada; 3 Sherbrooke Molecular Imaging Center, CRCHUS, Sherbrooke, QC Canada; 4 Department of Radiological Technology and Medical Physics, Faculty of Allied Health Sciences, Chulalongkorn University, Bangkok, Thailand

**Keywords:** ^18^F-fluorothymidine (^18^F-FLT), 5-Fluorouracil (5FU), Intratumoral (*i.t.*) infusion, Targeted radiotherapy (TRT), 2-deoxy-2-[^18^F]-fluoro-D18 glucose (^18^F-FDG), Positron emission tomography (PET)

## Abstract

**Background:**

The goal of targeted radiotherapy (TRT) is to administer radionuclides to tumor cells, while limiting radiation exposure to normal tissues. 3′-Deoxy-3′-[^18^F]-fluorothymidine (^18^F-FLT) is able to target tumor cells and emits a positron with energy appropriate for local (~ 1 mm range) radiotherapy. In the present work, we investigated the potential of TRT with a local administration of ^18^F-FLT alone or in combination with 5-fluorouracil (5FU), which acts as a chemotherapeutic agent and radiosensitizer. Treatment efficiency of ^18^F-FLT combined or not with 5FU was evaluated by intratumoral (*i.t.*) infusion into subcutaneous HCT116 colorectal tumors implanted in nu/nu mice. The tumor uptake and kinetics of ^18^F-FLT were determined and compared to 2-deoxy-2-[^18^F]-fluoro-D-glucose (^18^F-FDG) by dynamic positron emission tomography (PET) imaging following *i.t.* injection. The therapeutic responses of ^18^F-FLT alone and with 5FU were evaluated and compared with ^18^F-FDG and external beam radiotherapy (EBRT). The level of prostaglandin E_2_ (PGE_2_) biosynthesis was measured by liquid chromatography/tandem mass spectrometry (LC/MS/MS) in order to determine the level of inflammation to healthy tissues surrounding the tumor, after *i.t.* injection of ^18^F-FLT, and compared to EBRT.

**Results:**

We found that *i.t.* administration of ^18^F-FLT offers (1) the highest tumor-to-muscle uptake ratio not only in the injected tumor, but also in distant tumors, suggesting potential for concurrent metastases treatment and (2) a sixfold gain in radiotherapeutic efficacy in the primary tumor relative to EBRT, which can be further enhanced with concurrent *i.t.* administration of the radiosensitizer 5FU. While EBRT stimulated PGE_2_ production in peritumoral tissues, no significant increase of PGE_2_ was measured in this area following *i.t.* administration of ^18^F-FLT.

**Conclusion:**

Considering the biochemical stability of ^18^F-FLT and the physical properties of localized ^18^F, this study shows that TRT via intratumoral infusion of ^18^F-FLT and 5FU could provide a new effective treatment option for solid tumors. Using this approach in a colorectal tumor model, the tumor and its metastases could be efficiently irradiated locally with much lower doses absorbed by healthy tissues than with *i.t.* administration of ^18^F-FDG or conventional EBRT.

**Electronic supplementary material:**

The online version of this article (10.1186/s13550-019-0496-7) contains supplementary material, which is available to authorized users.

## Background

Positron emission tomography (PET) is capable of assessing tumor proliferation quantitatively, non-invasively, and reproducibly [1]. Proliferation is a key feature of tumor progression and the principal mechanism underlying 3′-deoxy-3′-[^18^F]-fluorothymidine (^18^F-FLT) PET imaging [2, 3]. ^18^F-FLT crosses the cell membrane via specific nucleoside transporters [4]. The principal mechanism underlying ^18^F-FLT PET imaging is the uptake by proliferating cells of the tracer into the pyrimidine exogenous salvage pathway or endogenous de novo pathway. Aside from phosphorylation in proliferating cells, the compound is metabolically stable in vivo, ^18^F is not released from FLT and hence the free atom poses no risk of accumulation in sensitive tissues [5]. However, selective uptake of FLT in the bone marrow, a tissue with a high proliferative rate, has considerably limited investigations on its potential use as a theranostic agent administered intravenously (*i.v.*) [6].

Similar to thymidine, ^18^F-FLT once internalized into the cytoplasm is phosphorylated into ^18^F-FLT monophosphate by thymidine kinase 1 (TK1) that has the particularity to become trapped inside the cell without being incorporated into the DNA [7]. Alternatively, in the de novo synthesis pathway, deoxyuridine monophosphate is converted to thymidine monophosphate by thymidine synthase which can be phosphorylated and incorporated into DNA [8]. The accumulation of ^18^F-FLT is dependent on the presence of TK1, which is closely associated with cellular proliferation [7], and poor prognosis for cancer patients [9]. ^18^F-FLT has therefore the unique potential to preferably target this group of poor prognosis cancers. Preclinical and clinical studies have demonstrated a considerable interest in ^18^F-FLT as a PET tracer in breast, lung, and brain cancer imaging [4, 7, 10–12]. ^18^F-FLT PET has been previously shown to provide valuable information for response assessment of tumor therapies [3, 7], and it has found limited use for tumor therapy follow-up in clinical trials [8, 13]. ^18^F-FLT tumor uptake is generally lower than that of 2-deoxy-2-[^18^F]-fluoro-D-glucose (^18^F-FDG), but its selectivity for tumor versus inflammatory cells often makes it a better marker of tumor cells than the glucose analog, ^18^F-FDG [7]. Moreover, ^18^F-FLT showed low uptake in the brain and heart, in contrast to ^18^F-FDG, confirming its higher tumor specificity [14]. The only limitation remains the detection and measurement of bone tumors and metastases, due to the high ^18^F-FLT uptake in healthy bone marrow.

Positron-labeled radionuclides are beginning to be widely used in combined diagnostic and therapeutic applications, which are also known as the theranostic feature of nuclear medicine [15]. The average kinetic energy of the positron emitted by ^18^F is about a quarter of that generated by its annihilation [11]. However, due to the physical properties of ^18^F radioactive decay, targeted radionuclide therapy (TRT) by emission of the positron is expected to be highly effective, since radiation damage from positrons can be localized within ~ 1 to 2 mm [11] from their source. On the other hand, the 511 keV photons from annihilation, which provide the tomographic images, have almost a three orders of magnitude longer penetration depth in tissues [16]. These physical characteristics imply that, in TRT, the dose densities absorbed by the body are negligible compared to that absorbed locally in the targeted cancer cells (i.e., radiation flux decreases as the square of the distance from the annihilation point), unless some tissues express a particularly high uptake, such as the bone marrow [6] after *i.v.* administration.

Moadel et al. investigated cancer cell affinity of PET radiotracers. They have shown, for the first time at a cellular level, the radiotherapeutic potential of *i.v.* administration of ^18^F-FDG [17, 18]. Although ^18^F-FDG significantly slowed tumor growth and prolonged survival in comparison with non-treated animals, high cardiac and brain uptakes, as well as an important renal radiotoxicity, were observed, partially offsetting the benefits of this novel approach. This barrier to such a theranostic clinical application could be overcome to a large extent by ^18^F-FLT. The latter has been developed to accumulate specifically in tumors, proportionally to the proliferation rate of the active cells [2, 3], and showed low uptake in the brain and heart, compared to ^18^F-FDG [14]. Moreover, utilizing ^18^F-FLT in cancer treatment not only allows improving the determination of tumor prognosis and monitoring tumor response to anti-cancer treatment, but also may offer some advantages of a positron-labeled radiopharmaceutical as a therapeutic agent. Combined with *i.t.* injection, these benefits may partly offset the dose delivered to the bone marrow by ^18^F-FLT.

Here, we investigate the potential of metabolic TRT with ^18^F-FLT in an animal model of human colon cancer derived from HCT116 cells, which are known to be highly proliferative, when implanted in immuno-deficient mice [19]. Considering that the therapeutic properties of ^18^F-FLT may be dependent on the mode of administration, we explore the biodistribution by PET and TRT of ^18^F-FLT in mice, after direct infusion of the radioactive compound in a primary tumor by convection-enhanced delivery (CED) [20]. ^18^F-FLT was administered directly into one tumor, while contralateral tumors simulated distant metastases. As a predictive tool in assessing radiotherapy efficacy, tumor uptake was monitored by PET following ^18^F-FLT and ^18^F-FDG *i.t.* infusion under identical conditions. The results were compared to those obtained from a single 15 Gy dose of external beam radiotherapy (EBRT), considered equivalent to the 25 fractions of 2 Gy [21] frequently used in cancer treatment. To highlight the full potential of *i.t.* ^18^F-FLT treatment, such comparisons were also made in combination with the chemotherapeutic agent 5FU.

## Methods

### ^18^F-FDG and ^18^F-FLT

^18^F-FDG (CYCLODX, CIUSSS de l’Estrie - Centre Hospitalier Universitaire de Sherbrooke, Canada) and ^18^F-FLT were prepared by the Sherbrooke Molecular Imaging Center (CIMS, Sherbrooke, Quebec, Canada). ^18^F-FLT was produced using the protected nosylate precursor and the method of Yun et al. [22].

### Cell culture

The HCT116 human colorectal carcinoma cell line obtained from ATCC was routinely cultured in modified Eagle’s medium (Sigma-Aldrich, Oakville, Canada) supplemented with 10% fetal bovine serum, 2 mM glutamine,1 mM sodium pyruvate, 100 units/ml penicillin, and 100 μM streptomycin in a fully humidified incubator at 37 °C in an atmosphere containing 5% CO_2_.

### Human colorectal cancer xenograft mouse model

Experiments were performed with outbred male nude mice at 4–6 weeks of age (Charles River Laboratories, Saint-Constant, QC, Canada). The animals were maintained in an animal facility, under specific pathogen-free conditions. Housing and all procedures involving animals were performed according to the protocol approved by the Université de Sherbrooke Animal Care and Use Committee (protocol number 235-14B). Human colorectal HCT116 tumor cells (2 × 10^6^, 0.1 mL) were inoculated subcutaneously (*s.c.*) into each rear thigh and one on the right shoulder. During each animal handling implantation, the animals were anesthetized with an intraperitoneal injection of ketamine/xylazine (87/13 mg/mL) at 1 mL/kg. Tumor size measurements began 1-week post-injection and continued biweekly. Tumor volumes were calculated with the following formula: *V* (mm^3^) = π/6 × *a* (mm) × *b*^2^ (mm^2^), where *a* and *b* were the largest and smallest perpendicular tumor diameters, respectively. All experiments began when tumor volumes reached a diameter of about 5–7 mm. The tumor-bearing animals were randomized into different groups of two to four animals each.

### Distribution kinetics and tumor clearance of *i.t.*^18^F-FLT and ^18^F-FDG

The animals were anesthetized by inhalation of 1.5% isoflurane and 1.5 L/min oxygen during *i.t.* injection and PET imaging procedures. The single *i.t.* infusion of 5 MBq of ^18^F-FLT or ^18^F-FDG solution was applied into the tumor on one side of the rear thigh, whereas the contralateral tumors were not treated. The solution was introduced into the central section area of the tumor. For each injection, the needle tip placement was at approximately one-third depth in the tumor along with the needle insertion direction. The leakage of the radiolabeled compound is the main concern for *i.t.* injection. To avoid this complication, the *i.t.* infusion was performed at a slow infusion rate (10 μL/min) over 10 min and the needle was left in place within the tumor for about 5 min following completion of the *i.t.* infusion to reduce any backflow of the ^18^F-FLT or ^18^F-FDG solution. Total infusion volume for each tumor was limited to about 30–50% of the tumor volume from caliper measurements, which were determined on the day of the study.

The administration of ^18^F-FLT or ^18^F-FDG by *i.t.* injection was performed with the animal placed inside the scanner, at the start of data acquisition at time 0. Dynamic PET data were acquired in list mode from time 0 to 120 min post-injection using the Triumph/LabPET8™ platform (Gamma Medica, Northridge, CA) at the CIMS.

PET images were reconstructed on a 120 × 120 × 128 matrix with a 0.5 × 0.5 × 0.6 mm^3^ voxel size using the standard LabPET 3D maximum likelihood expectation maximization algorithm implementing a 3D model of the physical detector response. Frame durations for the reconstructed images were 10 × 1 min, 10 × 5 min, and 4 × 15 min. All PET images were corrected for physical radionuclide decay, dead time, and differences in crystal detection efficiency.

To quantify the radiotracer uptake, regions of interest (ROI) were drawn around tumors, organs, and whole body in the last image frame using the Amide software [23]. These ROIs were then applied to all frames to obtain time-activity curves (TAC) for each organ. The ROI activity was expressed as percent injected dose per gram of tissue (%IA/g) with the whole body radioactivity measured by PET. The residency time (in hours) for each organ was calculated using decay-uncorrected TAC as follows [24]:

$$ {\tau}_{\mathrm{h}}=\frac{\underset{0}{\overset{2\mathrm{h}}{\int }}\mathrm{TAC}(t) dt+\mathrm{TAC}\left(2\mathrm{h}\right)\underset{2\mathrm{h}}{\overset{\infty }{\int }}{e}^{-\lambda t} dt}{A_0} $$

where TAC*(t)* is the activity in the organ at time *t*, TAC(2 h) is the activity in the organ at the last time point of measurement (2 h), *λ* is the physical decay constant of ^18^F, and *A*_0_ is the injected activity in the main tumor. Trapezoidal rule was used to numerically integrate the organ TAC over the measurement time, while the analytical integration was performed on the exponential decay term.

In order to assess the radiation burden of ^18^F-FLT to bone and bone marrow, the uptake of ^18^F-FLT following *i.t.* (*n* = 3) and *i.v.* (*n* = 1) administration of ^18^F-FLT was compared from PET acquisitions by tracing ROI on the forepaw long bones and on the spine. The relative exposure of the bone marrow was estimated from the area under the non-decay-corrected time-activity curves (AUC) extrapolated to infinity with the physical decay of ^18^F.

### Determination of the tumor response after *i.t.* infusion of ^18^F-FLT and ^18^F-FDG radiotherapy and EBRT

In order to determine the dose dependence of tumor response, single *i.t.* injections of 15 and 25 MBq ^18^F-FLT or ^18^F-FDG were administered into the tumor on one side of the rear thigh, whereas the contralateral tumors were left untreated. All experiments began when tumor volumes reached a diameter of about 5–7 mm.

External beam gamma radiation was performed with a 4C Gamma Knife (Elekta Instruments AB, Stockholm, Sweden). A single *i.t.* injection of 0.9% saline was administered into the tumor to emulate the radiotracer administration. Mice were anesthetized and positioned in our in-house stereotactic frame designed for the 4C Gamma Knife [25]. The radiation treatment (15 Gy, dose rate of 3.6 Gy/min) using 8-mm collimators was delivered at predetermined coordinates targeting the tumor. Radiation was applied to the tumor located on one side of the rear thigh, whereas the other side was kept as the non-irradiated control tumor.

Tumor growth was measured after treatment twice a week. Tumor volumes were calculated as described in the mouse model section. Fivefold growth delay (5Td) was considered to be the time required for the tumor volume to increase by a factor of 5, compared to the initial volume at the beginning of treatment. Tumor growth delay (TGD) was calculated by subtracting the 5Td value of the treated group from the 5Td of the control group. An enhancement factor (EF) was also calculated by dividing the 5Td of the treated group by the 5Td of the non-treated control group.

#### Assessment of absorbed dose from PET imaging by Fricke dosimetry

The mean ^18^F-FDG activity in different tissues measured in our mouse model by PET imaging was correlated to the absorbed dose assessed in vitro by Fricke dosimetry [26, 27], as described by Tippayamontri et al. [28]. Briefly, the dose-response of the Fricke dosimeter and total activity measured by PET were determined at different times for a 3 mL Fricke solution and a 3 mL of deionized water that contained 60 MBq of ^18^F-FDG. The total absorbed dose in the Fricke solution was assessed at 1450 min. The dose was correlated to the activity measured by PET for the same solution. The characteristics of the LabPET8™ scanner (Gamma Medica) was previously described in [29]. During the calibration procedure, PET imaging was performed at time 0, 0.5, 1, 2, 3, and 4 h after adding ^18^F-FDG into the Fricke solution, with scanning times of 3, 5.12, 9.50, 16.24, and 30.06 min, respectively. The optical density and radioactivity were measured prior to and after the PET scans. CT imaging of the vials was performed for attenuation correction of the emission data. The raw data were reconstructed and corrected relative to the reconstructed resolution of the PET scanner. The decay, dead time, random subtraction, and differences in crystal detection efficiencies were also included in the correction factor. A region of interest (ROIs) analysis was carried out with the built-in function in the LabPET image analysis software. The radioactivity in the subject vial (Fisherbrand 15 × 45 mm, 1DR, Fisher Scientific) was obtained as cps/mL from reconstructed PET images. The relationship of absorbed dose (Gy) and time-integrated activity (MBq.h) with administered activity (MBq) is shown in the Additional file 1: Figure S1 and Additional file 2: Figure S2, respectively.

To obtain quantitative radioactivity data with mice, the PET system was calibrated by acquiring data from a mouse phantom filled with an ^18^F-FDG solution of known radioactivity. Thus, the pixel counts of the PET image in cps/mL could be converted into the activity concentration (MBq/mL) by multiplying the ROIs with known added activity of ^18^F-FDG. Total accumulated absorbed dose in the tumor tissue and normal organs can be calculated by following Eq. 1.

1 $$ D\left(\mathrm{Gy}\right)=\breve{A}\left(\mathrm{MBq}.\mathrm{h}/\mathrm{g}\right)\times M\ \left(\mathrm{g}\right)\times C\left(\mathrm{Gy}/\mathrm{MBq}.\mathrm{h}\right) $$

where:

*Ă* is the time-integrated activity per gram of tissue (MBq.h/g)

*M* is the tissue mass (g)

*D* is the absorbed dose (Gy)

*C* is the conversion factor of 0.09 Gy/MBq.h, derived from the relationship between Fricke dosimetry and PET imaging (Additional file 3: Conversion factor for absorbed dose estimated by the Fricke chemical primary standard dosimeter).

### Prostaglandin E_2_ quantification by liquid chromatography/tandem mass spectrometry

PGE_2_ has been quantified to assess inflammation [30]. Muscle tissues nearby the irradiated area were extracted and snapped frozen with liquid nitrogen after 4 h of either *i.t.* injection of 5 MBq ^18^F-FLT or 15 Gy gamma irradiation. Tissues were homogenized with a Dounce homogenizer in 2 mL of acetone-saline solution (2:1), containing 10 ng of the internal standard prostaglandin E_**2**_d_**4**_ (PGE_**2**_-d_**4**_), which contains four deuterium atoms at the 3, 3′,4, and 4′ positions (internal standard, Cayman Chemical, Ann Arbor, MI, USA) and 0.05% butylated hydroxytoluene to prevent the oxidation of prostanoids. The homogenate was transferred to a screw-top tube, vortexed for 1 min, and centrifuged (10 min, 1800 g, room temperature). The supernatant was transferred to another tube and mixed with 2 mL hexane by vortexing for 1 min. After centrifugation (10 min, 1800 g, room temperature), the upper phase containing lipids was discarded. The lower phase was acidified with 30 μL of 2 M formic acid and then 2 mL of chloroform containing 0.05% butylated hydroxytoluene were added. The mixture was vortexed and again centrifuged (10 min, 1800 g, room temperature) to separate the two phases. The lower phase containing chloroform was transferred to a conical centrifuge tube for evaporation with a SpeedVac Concentrator (Sarant, Nepean, ON, Canada). Samples were reconstituted in 100 μL methanol:10 mM ammonium acetate buffer, pH 8.5 (70:30), and filtered with Spin-X centrifuge tube filter 0.45 μm (10 min, 1300 g, room temperature). Samples were stored at − 20 °C for later analyses.

PGE_**2**_ was quantified by LC/MS/MS using the same procedure as reported by Desmarais et al. [31]. Briefly, the apparatus consisted of an API 3000 mass spectrometer (Applied Biosystem, Streesville, ON, Canada) equipped with a Sciex turbo ion spray (AB Sciex, Concord, ON, Canada) and a Shimadzu pump and controller (Columbia, MD, USA). Prostaglandins were chromatographically resolved using a Kromasil column 100-3.5C18, 150 × 2.1 mm (EKa Chemicals, Valleyfields, QC, Canada). A linear acetonitrile gradient from 45 to 90% during 12 min at a flow rate of 200 μL/min was used. The mobile phase consisted of water buffered with 0.05% acetic acid and acetonitrile 90% with acetic acid 0.05%. The injection volume was 10 μL per sample, which were kept at 4 °C during analysis. Individual products were detected using negative ionization and the monitoring of the transition m/z 351 ➔ 271 for PGE_**2**_ and 355 ➔ 275 for PGE_**2**_d_**4**_ with a collision voltage of − 25 V. For quantification of specific ions, the area under the curves was measured.

### Mitotic activity assessed by immunohistochemistry of Ki67

Animals were euthanized 4 h after combined *i.t.* treatment with 5FU and 5 MBq ^18^F-FLT or 15 Gy gamma irradiation. Tumor samples were removed and fixed in 10% buffered formalin. The 5-μm sections from paraffin-embedded blocks were stained with conventional hematoxylin-eosin.

For Ki67 staining, 5-μm sections from paraffin-embedded blocks were deparaffinized in xylene, rehydrated using graded alcohol, and washed with PBS buffer (pH 7.4). For antigen retrieval, sections were placed in 0.01 M sodium citrate buffer (pH 6.0) for 10 min inside a steamer cooker. Sections were cooled to room temperature and washed with PBS buffer. Endogenous peroxide was blocked by 3% H_2_O_2_ for 15 min. Sections were incubated in 10% bovine serum albumin (BSA) for 1 h at room temperature. Thereafter, sections were incubated in overnight at 4 °C with the primary mouse monoclonal antibody Ki67 diluted in 0.5% BSA (PM375 AA Biocare Medical, Concord, California, USA). Sections were treated with the second anti-rabbit antibody (PM375 AA Biocare Medical, Concord, California, USA) diluted in 0.5% BSA for 1 h at room temperature. Diaminobenzidine tetrahydrochloride (0.6 mg/mL in Tris buffer saline, pH 7.6 containing 0.04% hydrogen peroxide) was used to develop the brown color. Methyl green was used to counterstain the slides. A negative control (with primary antibody omitted) was taken along with each batch. Counting of Ki67-positive cells was carried out in ten consecutive fields of 20×. The Ki67 index was estimated by the percentage of Ki67-positive cells in all the counted tumor cells.

### Statistical analysis

All statistical analyses were performed using Prism 7.03 for Windows (GraphPad software). All results are reported as mean ± SD. The number of animals ranged from 2 to 4: (control untreated, *i.t.* 5FU, 15 Gy EBRT, *i.t.* 5FU + 15 Gy, *i.t.* 5FU + *i.t.* ^18^F-FLT 15 MBq, *i.t.* 5FU + *i.t.* ^18^F-FDG 15 MBq, *n* = 4), (PET *i.t.* ^18^F-FLT, *i.t.* FLT, *i.t.* ^18^F-FDG 15 MBq, untreated distant tumor (*i.t.* ^18^F-FLT 10 MBq), *n* = 3), and (PET *i.t.* ^18^F-FDG, *i.t.* ^18^F-FLT 15 MBq, *i.t.* ^18^F-FLT 25 MBq, *n* = 2). Statistical analyses were performed as described in the figures and table legends. Ordinary one-way ANOVA with a Dunnett’s multiple comparisons test was used to compare the residence time of ^18^F-FLT in the infused tumor to that of non-target tissues and to compare the 5Td of the non-treated animal to that of the different experimental groups. Ordinary one-way ANOVA with a Tukey’s multiple comparison test was used to compare the 5TD values of the (*i.t.* 5FU + 15 Gy EBRT) to those of the (*i.t.* 5FU + *i.t.* ^18^F-FLT 15 MBq) and (*i.t.* 5FU + *i.t.* ^18^F-FDG 15 MBq) groups. Differences were considered statistically significant at *p* ≤ 0.05.

## Results

### Distribution kinetics and clearance of *i.t.*^18^F-FLT and ^18^F-FDG

**Fig. 1:**
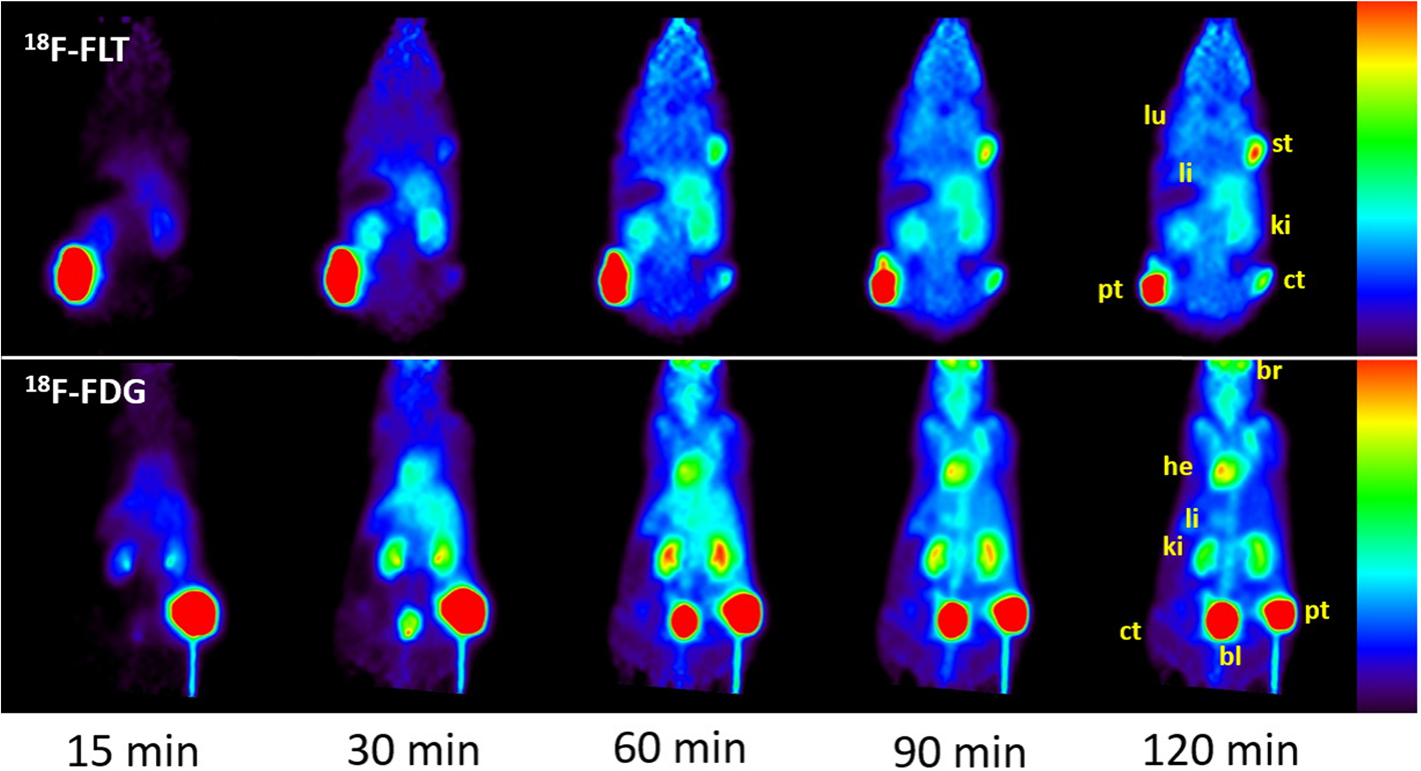
Kinetics of ^18^F-FLT (top) and ^18^F-FDG (bottom) distribution up to 120 min after *i.t* infusion of 5 MBq in the primary tumor (pt) of nude mice bearing the colorectal HCT116 tumor as assessed by PET imaging. Tumors were implanted on each thigh and additionally on the right shoulder solely for animals injected with ^18^F-FLT. ^18^F-FLT uptake increased steadily in the distant contralateral (ct) and shoulder tumors (st) as it decreased in the *i.t.* injected tumor, in contrast to ^18^F-FDG that mostly diffused in the heart (he), brain (br), and kidneys (ki). Lower accumulation in healthy tissues was obtained with *i.t.* ^18^F-FLT. lu denotes the lung and li denotes the liver

PET images of mice having received 5 MBq *i.t.* infusion over 10 min of ^18^F-FLT or ^18^F-FDG are displayed in Fig. 1. Comparison of the two sets of figures clearly shows that while primary tumor uptakes are similar for both tracers, uptakes in healthy organs are significantly reduced by *i.t.* administration of ^18^F-FLT. It is also worth noting that ^18^F-FLT activity grows steadily in the distant contralateral (non-injected) tumors, while that in the *i.t.* injected tumor decreases (Fig. 1). This is in contrast to ^18^F-FDG activity, which is eliminated from the primary tumor to multiple other tissues, without significant apparent accumulation in the contralateral tumor.

**Fig. 2:**
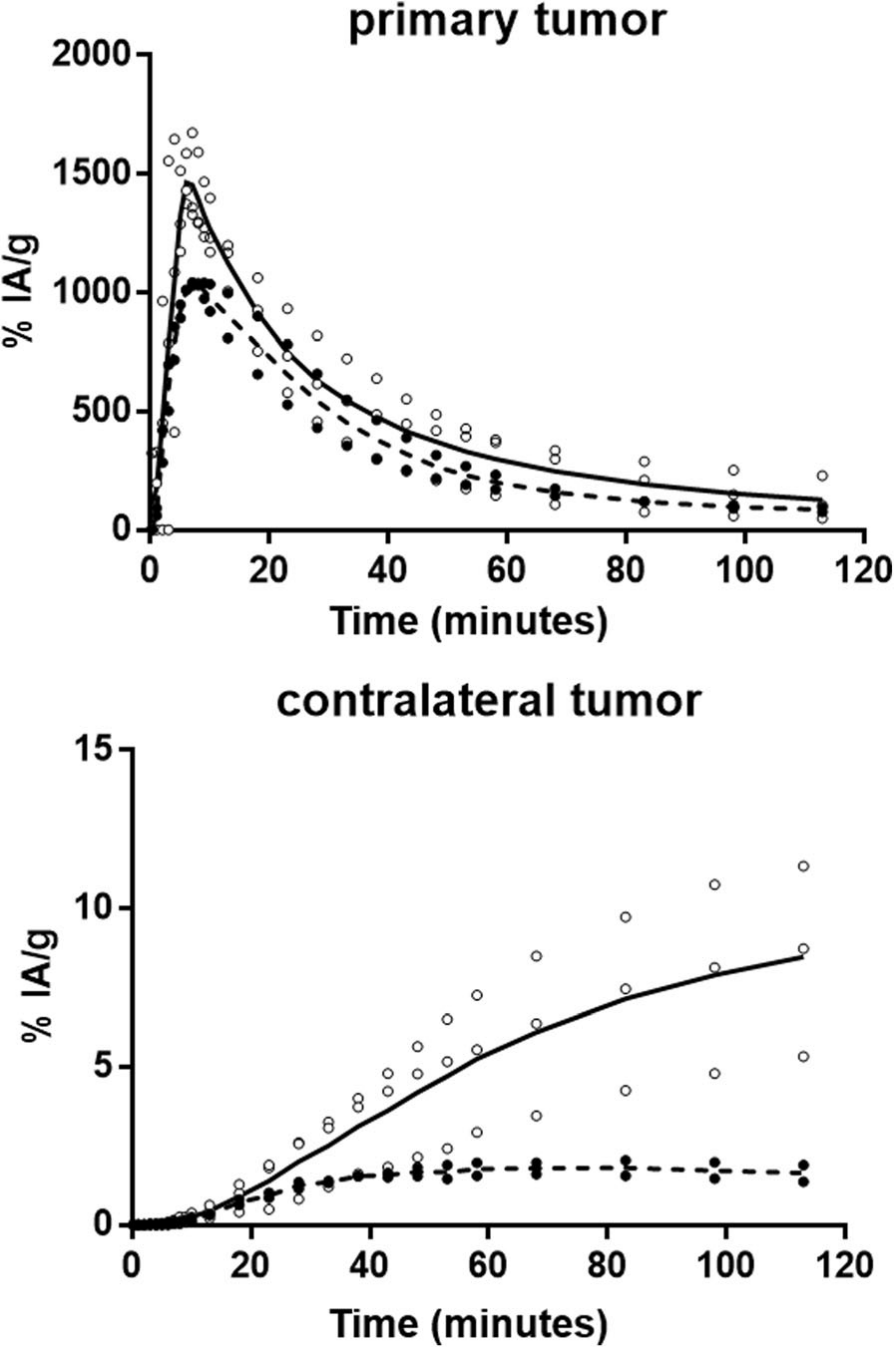
Tumor uptake and clearance of ^18^F-FLT (solid line) and ^18^F-FDG (dashed line) in mice bearing HCT116 human colorectal tumors after *i.t.* infusion of 5 MBq into the left rear flank tumor. The radiotracer kinetics was measured during 2 h by dynamic PET imaging. Data are expressed as the percent injected activity per gram for tissue (%IA/g). All data were corrected for physical decay of the isotope

**Fig. 3:**
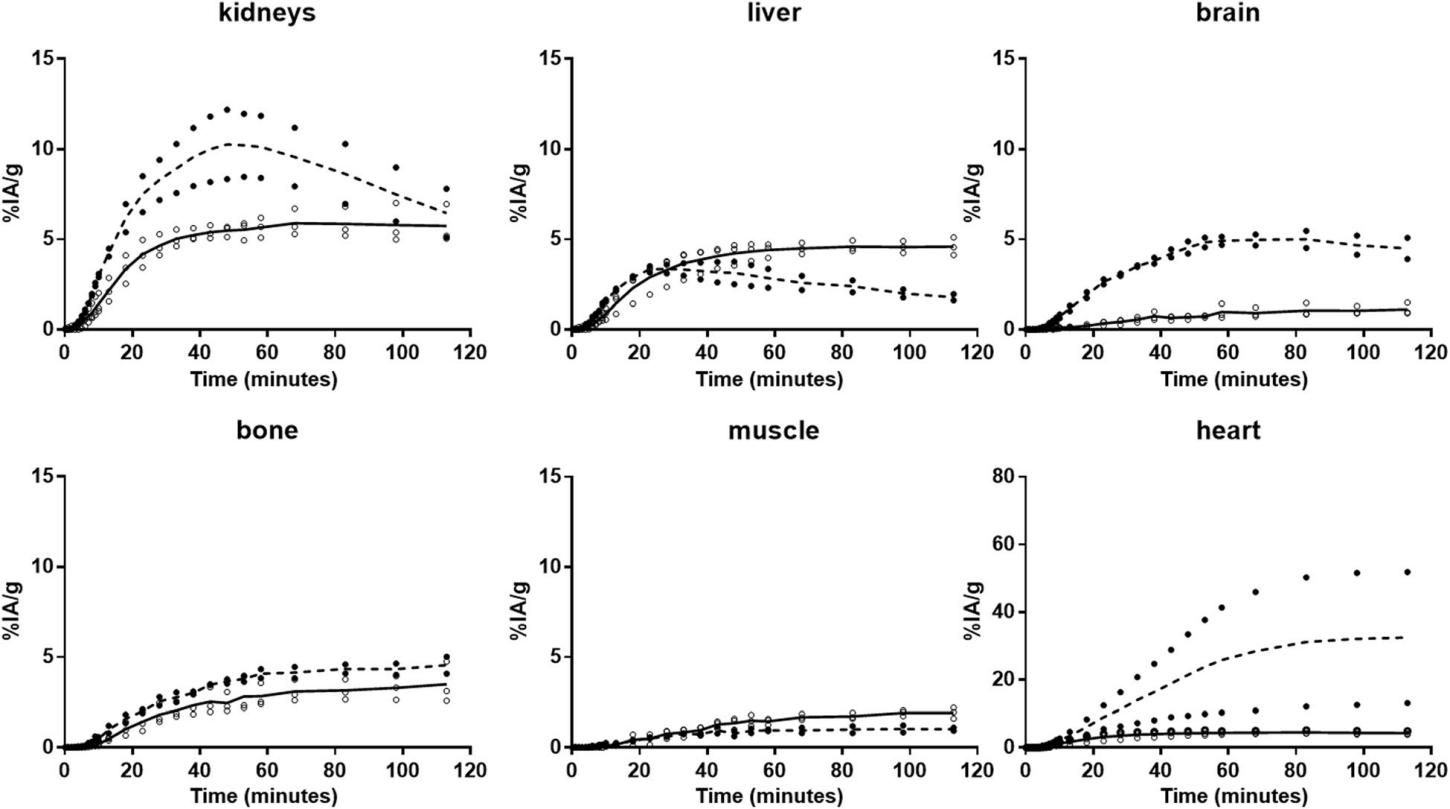
Distribution kinetics and clearance of ^18^F-FLT (solid line) and ^18^F-FDG (dashed line) in mice bearing HCT116 human colorectal tumors after *i.t.* infusion of 5 MBq into the rear flank tumor. The radiotracer biodistribution kinetics were measured during 2 h by dynamic PET imaging. Data are expressed as the percent injected dose per gram of tissue (%IA/g). All data were corrected for physical decay of the isotope

Radiotracer uptake in the *i.t.* injected tumor reaches a maximum of about 10 min after the beginning of the infusion and drops asymptotically to less than 10% of the maximum after 120 min (Fig. 2). At this time, contralateral proximal and distant tumors showed similar ^18^F-FLT uptakes of ~ 8%IA/g. The ^18^F-FDG kinetics follow a different trend in the contralateral tumor, reaching a plateau at 40 min and a tumor retention only about one fifth that of ^18^F-FLT at 120 min (Fig. 2). As observed in Fig. 1, time-activity curves corroborate the significantly lower and slower uptakes in the kidneys, brain, bone, and heart with *i.t.* infusion of ^18^F-FLT compared to ^18^F-FDG (Fig. 3).

**Fig. 4:**
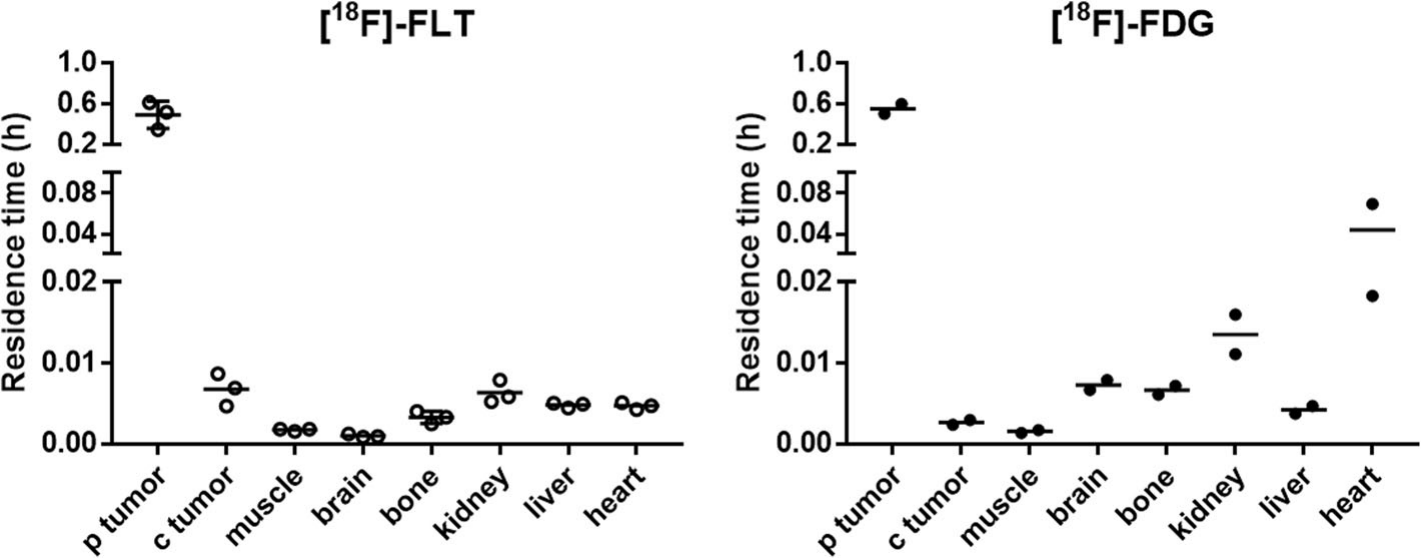
Residence time of ^18^F-FLT and ^18^F-FDG in each tissue. Residence times were calculated as areas under the time-activity curves shown in Figs. 2 and 3 extrapolated to infinity with the physical decay of ^18^F. The horizontal bars stand for the mean

The residence time of ^18^F-FLT and ^18^F-FDG in each tissue is displayed in Fig. 4. For ^18^F-FLT, the contralateral tumor residence time is significantly higher than those of the muscle, bone, and brain. By contrast, the largest residence time in non-target tissues for ^18^F-FDG was found in the brain, bone, kidneys, and heart.

**Fig. 5:**
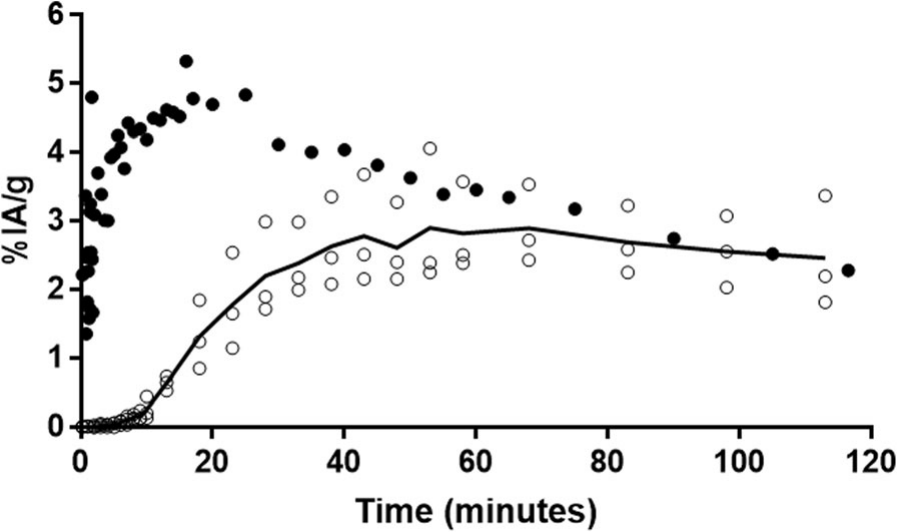
Bone uptake of [^18^F]-FLT following *i.v.* (full circles) or *i.t.* (solid line with open circles, n=3) administration. Data obtained from ROI traced on the forepaw bones and spine. The data are not corrected for ^18^F decay

The time-activity curves for *i.t.* and *i.v.* administration of ^18^F-FLT are presented in Fig. 5 for the data not corrected for radioactive decay. Bone uptake is much slower following *i.t.* administration as compared to *i.v.*, although both time-activity curves converged at 120 min post administration. The extrapolated AUC of these time-activity curves indicate a trend towards lower radiation burden, although there was no statistically significant difference (one sample *t* test) between the AUC (*i.t.* 5.12, sdm 1.37, *n* = 3; *i.v.* 6.7, *n* = 1) obtained with either methods of administration.

### Tumor response after local ^18^F-FLT *i.t.* infusion

**Fig. 6:**
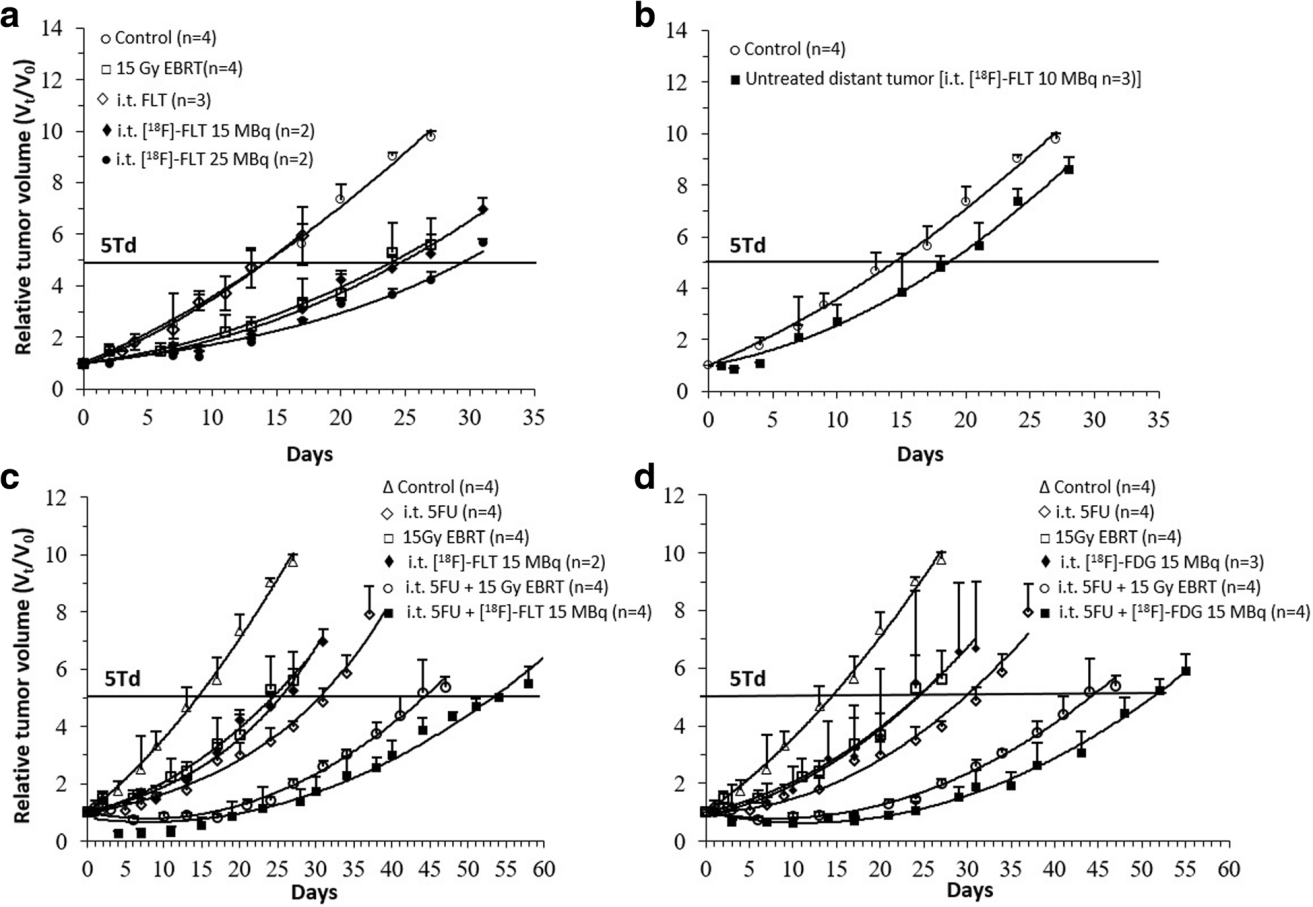
Tumor growth profiles after different treatment conditions in HCT116 colorectal xenografts compared to controls without treatment. The curves are normalized to the tumor volume at the beginning of follow up with **a** 15 Gy of external radiation and with different activities of *i.t.* injected ^18^F-FLT, **b** contralateral tumor after *i.t.* injected ^18^F-FLT, **c** combined treatment of *i.t.* 5FU and *i.t.* ^18^F-FLT or 15 Gy EBRT, and **d** combined treatment of *i.t.* 5FU and *i.t.* ^18^F-FDG or 15 Gy EBRT*.* The error bars represent the standard deviation on the mean for two to four animals

The therapeutic efficacy in animals treated with 15 and 25 MBq of *i.t.* ^18^F-FLT was compared to non-irradiated controls and (as a reference) to radiation delivered by a single 15 Gy dose EBRT (Fig. 6a). The therapeutic effect of ^18^F-FLT clearly derives from radiation damage as no reduction in tumor growth was found when non-radioactive FLT was injected (Fig. 6a). However, a significant improvement in tumor response was observed when using *i.t.* infusion of ^18^F-FLT, as evidenced in Table 1 by an increase in TGD and the EF. It is noteworthy that similar EFs were found with 15 MBq *i.t.* ^18^F-FLT (EF = 1.8), as with 15 Gy EBRT (EF = 1.7). Treatment effect can even be further enhanced with 25 MBq *i.t.* ^18^F-FLT (EF = 2.1, Table 1), at a dose still significantly lower than conventional radiotherapy. Furthermore, the unexpected uptake of ^18^F-FLT in the contralateral tumors with *i.t.* administration may be responsible for the slower growth rate compared to the control (Fig. 6b; EF = 1.3, Table 1), suggesting a potential role of *i.t.* ^18^F-FLT metabolic TRT in controlling distant metastases. The time-integrated activity was extracted specifically from the individual tumor tissues as well as normal organs. This therefore allows us to estimate the dose delivered to each tissue/organs from the ^18^F-radionuclide (Table 2), which can be further compared to the dose of 15 Gy delivered to the tumor by EBRT with the gamma knife. Animals treated with 15 MBq *i.t.* ^18^F-FLT received a lower radiation dose, for the same tumor treatment efficiency as with EBRT (Fig. 6a), while maintaining a low radiation exposure of healthy tissues (Fig. 3).

**Table 1:**
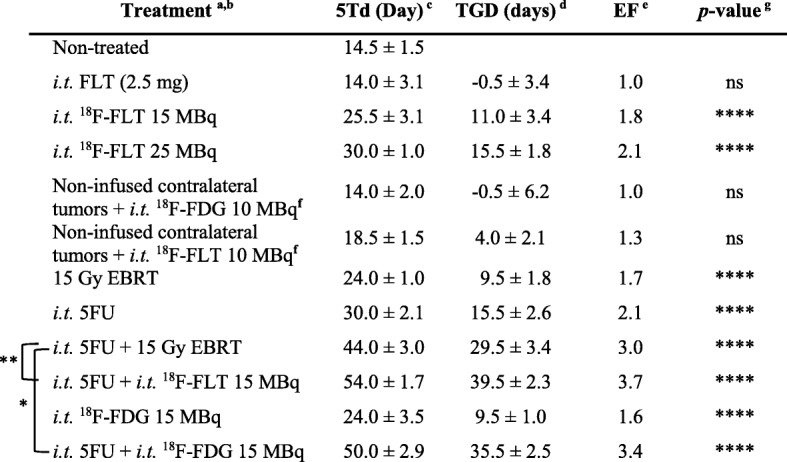
Therapeutic efficacy of ^18^F-metabolic TRT or EBRT, alone, or combined with *i.t.* 5FU in the HCT116 human colorectal cancer xenografts

^a^Control animals received 0.9% NaCl solution

^b^The single *i.t.* infusion of 2.5 mg 5FU was administered 4 h prior *i.t.* infusion of ^18^F-FLT or ^18^F-FDG, or of a single 15 Gy dose EBRT

^c^Five times tumor growth delay (5Td) was determined as the time required for the tumor volume to increase fivefold over the initial volume at the start of treatment

^d^Tumor growth delay (TGD) = 5Td of the treated group – 5Td of the non-treated group

^e^The enhancement factor (EF) = 5Td of the treated group/5Td of the non-treated group

^f^Growth of contralateral tumors after *i.t.* ^18^F-FLT 10 MBq into the primary tumor

^g^5TD *p* value of each treatment conditions in comparison with the control group, One-way ANOVA, *F*(10,27) = 130.2, *p* < 0.0001, Dunnett’s test and comparison of (*i.t.* 5FU + 15 Gy EBRT) with (*i.t.* 5FU + *i.t.* ^18^F-FLT 15 MBq) and (*i.t.* 5FU + *i.t.* ^18^F-FDG 15 MBq) Tukey’s multiple comparison test. ns: *p* > 0.05; *p ≤ 0.05; **p ≤ 0.01; ***p ≤ 0.001 ****p ≤ 0.0001. Two to four animals were included in each group

**Table 2:** Mean absorbed dose of ^18^F-FDG in different tissue/organs after *i.t.* ^18^F-FDG estimated by using the Fricke chemical primary standard dosimeter

Tissue	Mean absorbed dose (Gy)
Tumor	10.69 ± 0.12
Heart	1.91 ± 1.36
Liver	0.43 ± 0.40
Kidney	1.53 ± 0.55
Bladder	2.50 ± 1.67
Brain	0.29 ± 0.16

A single *i.t.* infusion of 5FU (2.5 mg/kg), 4 h prior to ^18^F-radiotherapy, results in a 2.1-fold better tumor growth inhibition than local radiotherapy alone (Table 1 and Fig. 6c, d). It is worth noting that treatment responses upon a combination of 5FU with 15 MBq *i.t.* ^18^F-FLT (EF = 3.7, Table 1) or ^18^F-FDG (EF = 3.4) are quite similar as might be predicted by their tumor uptake measured by PET. Concomitant administration of 5FU with 15 MBq *i.t.* ^18^F-FLT is noticeably more efficient (*p* ≤ 0.01) than when 5FU is combined with 15 Gy EBRT (EF = 3.0, *p* ≤ 0.05, Table 1).

### Inflammation response after *i.t.*^18^F-FLT infusion

**Fig. 7:**
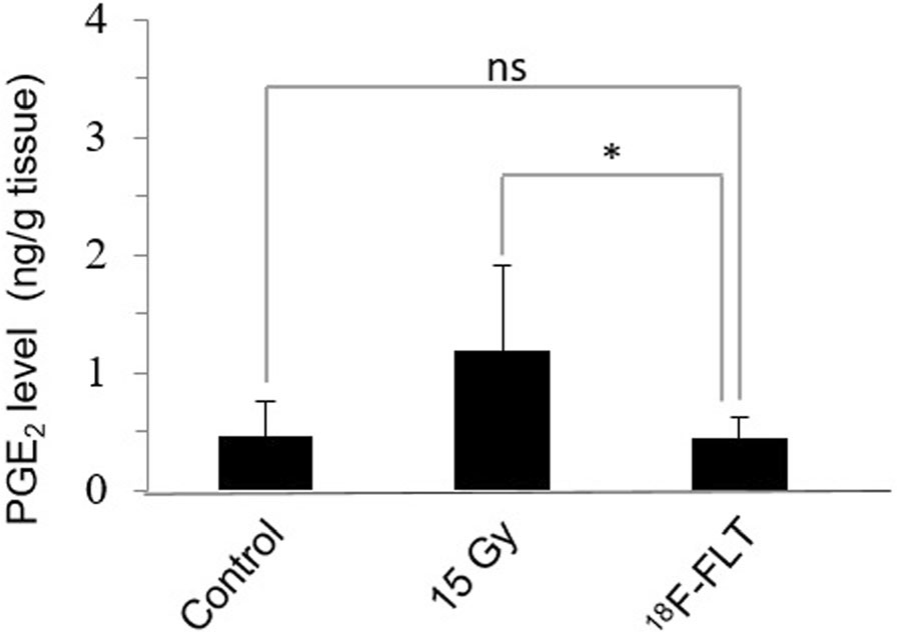
LC/MS/MS quantification of PGE_2_ in muscle tissues nearby irradiated tumors. Tumors received either 15 Gy gamma irradiation or *i.t.* injection of 5 MBq ^18^F-FLT. The animals were euthanized 4 h post-treatment. ns: *p* > 0.05; *p ≤ 0.05

PGE_2_ biosynthesis onto muscle tissues nearby the irradiated tumor areas was accessed 4 h post-treatment by LC/MS/MS (Fig. 7). After a single *i.t.* injection of ^18^F-FLT, the level of PGE_2_ was similar to that of the non-irradiated control group and about 2.5-fold lower than that measured in muscles tissues adjacent to the tumor having received a single irradiation of 15 Gy by EBRT.

### Tumor cell proliferation: Ki67 immunohistochemistry analysis

**Fig. 8:**
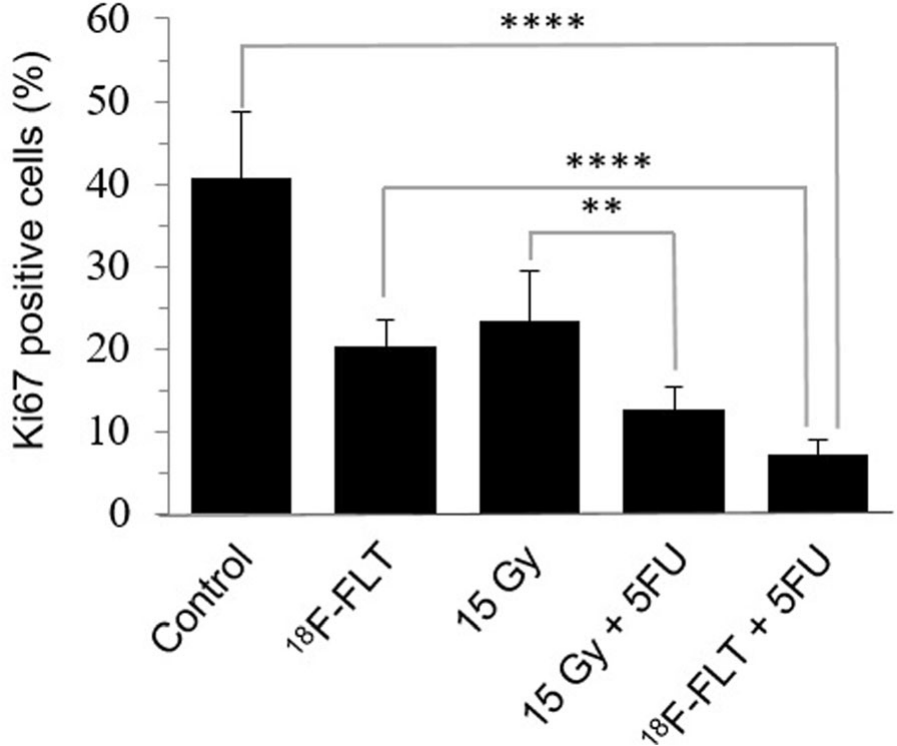
Immunohistochemical staining showing Ki67-based proliferation measured after treatment with *i.t.* ^18^F-FLT or 15 Gy EBRT, which were also combined with *i.t.* 5FU. ns: *p* > 0.05; *p ≤ 0.05; **p ≤ 0.01; ****p ≤ 0.0001

To further characterize the tumor response after combined *i.t.* 5FU with either 15 Gy EBRT or *i.t.* 5 MBq ^18^F-FLT in HCT116 xenograft tumors, the number of proliferating tumor cells was assessed by immunohistochemistry staining for Ki67 (Fig. 8). Tumors treated with 15 Gy EBRT alone (23.3 ± 6.2) or ^18^F-FLT alone (20.2 ± 3.4) showed a significant decrease in Ki67-positive cells compared to the control group (40.9 ± 7.9, *p* = 0.001). The proliferation index was further decreased when combining 15 Gy EBRT or *i.t.* ^18^F-FLT with *i.t.* 5FU radiosensitizers (12.8 ± 2.6 and 7.4 ± 1.7, respectively, *p* = 0.001).

## Discussion

The ratios in Table 3 provide a comparison of the radiospecificity of ^18^F-FLT and ^18^F-FDG relative to the kidneys, liver, brain, and heart. The distribution of ^18^F-FLT after *i.t.* injection could be followed by PET imaging in the primary tumor and the contralateral tumors, which simulated distant metastasis sites. We demonstrated that while uptakes were similar in the injected tumor following the *i.t.* ^18^F-FLT and ^18^F-FDG infusion, only ^18^F-FLT provided increased accumulation and residence time in distant tumors, coupled with low and slow uptakes in non-targeted organs. ^18^F-FLT demonstrated a higher specificity for the detection of primary and contralateral tumors than ^18^F-FDG following *i.t.* administration. Indeed, tumor-to-normal tissue ratios for the primary tumor were generally more favorable after *i.t.* infusion of ^18^F-FLT than with ^18^F-FDG (Table 3). More importantly, all tumor-to-tissue ratios for the contralateral tumors increased over time and were always superior to those of *i.t.* ^18^F-FDG, confirming a higher tumor specificity for ^18^F-FLT. One should note that the contralateral tumor ^18^F-FLT residence time, which is predictive of the therapy tumor-absorbed dose, significantly exceeded that of the muscle (*p* = 0.0001), brain (*p* ≤ 0.0001), and bone (*p* = 0.0034); it was also higher than that of the liver and heart, although the difference is not statistically significant for these tissues (Fig. 4). By contrast, the largest residence time for ^18^F-FDG was found in the brain, bone, kidneys, and heart, which are all particularly sensitive to ionizing radiation.

**Table 3:** Tumor-to-tissue ratios of ^18^F-FLT and ^18^F-FDG at three different times post injection after *i.t.* infusion of 5 MBq into a single tumor

Tissue	Time	Primary tumor		Contralateral tumor	
	(min)	^18^F-FLT	^18^F-FDG	^18^F-FLT	^18^F-FDG
Kidneys	30	111 ± 45	50.2 ± 4.5	0.49 ± 0.21	0.16 ± 0.04
	60	53 ± 25	19.9 ± 0.5	0.96 ± 0.48	0.18 ± 0.01
	120	23 ± 19	14.2 ± 6.3	1.56 ± 0.75	0.26 ± 0.02
Liver	30	162 ± 90	135 ± 21	0.66 ± 0.19	0.41 ± 0.07
	60	69 ± 33	71.4 ± 3.4	1.17 ± 0.41	0.63 ± 0.06
	120	26 ± 17	49 ± 14	1.84 ± 0.58	0.92 ± 0.08
Brain	30	1100 ± 656	129 ± 41	4.42 ± 1.21	0.39 ± 0.01
	60	334 ± 207	40.8 ± 6.3	5.37 ± 1.17	0.36 ± 0.04
	120	105 ± 50	19.8 ± 6.7	7.62 ± 2.04	0.36 ± 0.02
Heart	30	155 ± 88	46 ± 41	0.63 ± 0.18	0.13 ± 0.08
	60	70 ± 34	13.4 ± 13.1	1.18 ± 0.37	0.11 ± 0.11
	120	28 ± 17	3.8 ± 2.8	1.96 ± 0.58	0.09 ± 0.08

The ratios were determined from PET image-derived uptakes of the radiotracers

As expected, ^18^F-FLT bone uptake was lower at early times following *i.t.* infusion and similar to that of *i.v.* administration 2 h after (Fig. 5). However, the difference in radiation exposure as estimated from the area under the extrapolated curves is not statistically significant. Considering the low statistics involved, further studies are needed to evaluate the risk of radiation exposure of the bone marrow following metabolic TRT with ^18^F-FLT.

Currently, there is a growing interest in the use of nuclear medicine compounds not only for qualitative and quantitative evaluation of oncological pathologies, but also as treating agents [32]. The present investigations highlight the benefits of PET imaging to predict and measure the ^18^F-FLT treatment efficacy. The treatment with *i.t.* administration of ^18^F-FLT shows several key advantages compared to EBRT. The local radiation exposure associated with ^18^F-FLT arises from slowing down of the ^18^F-emitted positrons, with a mean energy of 250 keV, resulting in an average range in tissue of ~ 1 to 2 mm [11]. This latter property makes positron emission particularly suitable for local irradiation in the tumor tissue, while preventing damage to healthy tissues. The positrons lose their kinetic energy in tissue in the same manner as electrons [17], producing along their paths a considerable density of highly reactive ions and secondary low energy electrons [33]. In addition, “cross-fire” and “bystander” effects could potentiate tumor damage by such short-range positrons [34]. These unique properties of TRT may contribute to overcome the radio-resistance of solid tumors containing oxygen-deficient hypoxic, as higher radiation dose can potentially be delivered to tumor cells, while still preserving peritumoral healthy tissues.

The risk of radiation-induced late normal tissue injury, caused by chronic oxidative stress or inflammation, limits the dose of radiation that can be delivered safely to cancer patients [35]. In this study, we observed a low accumulation of *i.t.* administered ^18^F-FLT in healthy tissues and a low level of PGE_2_ and inflammation to healthy tissues surrounding the tumor. These data suggest that TRT with this radio-ligand has less off-target effects, making it more attractive for cancer radiotherapy. Moreover, *i.t.* infusion of ^18^F-FLT may allow higher doses of radiation to be locally delivered to target tissues than with EBRT, where the typical dose is in the range from 60 to 80 Gy [36]. The reported high selective uptake of ^18^F-FLT in the bone marrow represents a challenge for TRT using this radiopharmaceutical [6]. However, our results show that with an *i.t.* protocol, the radiation exposure is of the same order as for other sensitive tissues such as the brain, heart, and liver. It is also noteworthy that the radiation burden to bone and bone marrow is significantly lower than for *i.t.* administration of ^18^F-FDG.

Previous studies have demonstrated the therapeutic potential of positrons in the colon, breast carcinoma, and lung metastases upon *i.v.* or intraperitoneal (*i.p.*) injection of ^18^F-FDG [17, 37–39]. Fang et al. reported an improvement of tumor response in a mouse model, compared to a control group, in colon cancer treated with 55.5 to 222 MBq of ^18^F-FDG via *i.p.* administration. However, no significant difference between treated groups was observed [38]. In the present study, the dose range of ^18^F-FLT used in *i.t.* injection was an order of magnitude lower than in these earlier studies. Nevertheless, a significant tumor growth delay relative to the control group was observed after *i.t.* injection of 15 MBq ^18^F-FLT. Similar TGDs and EFs were observed for tumors treated with 15 MBq of ^18^F-FLT and those receiving 15 Gy EBRT. Despite a similar response to treatment, it is somewhat difficult to correlate these two results together. Our experiment with the Fricke dosimeter demonstrated that the radiation dose delivered to the tumor from the *i.t.* injection of 15 MBq of ^18^F-FLT (assuming that all the radiation energy of ^18^F-FLT remains in the tumor) is received lower than that from EBRT for the same biological effect. Therefore, our results suggest that a therapeutic benefit from direct exposure to positron emitting agents can be achieved with local ^18^F-radiotherapy.

TRT can be enhanced even further by the concurrent administration of the radiosensitizing agent 5FU [39]. Tumor cell proliferation assessed by Ki67 labeling index was evaluated based on previous studies [40] at 4 h post-combined treatment of *i.t.* 5FU with either *i.t.* injection of ^18^F-FLT or 15 Gy EBRT (Fig. 8). The concurrent *i.t.* administration of the chemotherapeutic agent 5FU with ^18^F-FLT reduced considerably tumor cell proliferation induced by ^18^F-FLT even at a suboptimal 5 MBq dose, as compared to the combined treatment of *i.t.* 5FU with 15 Gy radiation. Moreover, this combined treatment did not induce any body weight loss in mice at any doses (Table 4), indicating that it was well tolerated. Thus, adding 5FU to ^18^F-FLT could considerably enhance TGD and EF, without significantly increasing damage to healthy tissues, including the bone marrow.

**Table 4:** Change in body weight of nude mice after different treatment conditions

Treatment	Before Treatment (g)	10 Days after treatment (g)	Days to reach 5Td (g)
Control	27.0 ± 0.7	26.2 ± 2.4	29.9 ± 2.2
*i.t.* FLT	26.5 ± 1.5	27.2 ± 1.3	30.4 ± 1.6
*i.t.* ^18^F-FLT 15 MBq	30.0 ± 1.1	28.4 ± 0.5	32.7 ± 1.1
*i.t.* ^18^F-FLT 25 MBq	27.3 ± 2.2	26.7 ± 0.9	28.9 ± 2.2
15 Gy EBRT	25.8 ± 2.8	26.4 ± 0.5	27.9 ± 2.4
*i.t.* 5FU	26.5 ± 2.9	26.0 ± 2.8	29.5 ± 4.3
*i.t.* 5FU + 15 Gy EBRT	23.8 ± 3.4	25.6 ± 0.6	28.1 ± 1.2
*i.t.* 5FU + *i.t.* ^18^F-FLT 15 MBq	23.1 ± 0.3	23.6 ± 0.4	24.8 ± 1.0
*i.t.* ^18^F-FDG 15 MBq	27.3 ± 1.9	28.1 ± 1.6	29.9 ± 2.9
*i.t.* 5FU + *i.t.* ^18^F-FDG 15 MBq	30.6 ± 0.8	30.0 ± 1.7	32.3 ± 1.0
*i.v.* ^18^F-FLT 10 MBq	26.8 ± 1.9	27.6 ± 0.5	29.3 ± 0.6

Three to five animals were included in each group

Since local tumor recurrence and metastases are a major concern in cancer treatment, the fast transfer of ^18^F-FLT from the treated tumor to non-injected tumors may be a significant benefit after *i.t.* infusion (Fig. 1) via convection-enhanced delivery (CED) [20]. This is indicative of fast distribution and circulation rates for ^18^F-FLT through tumor vascularization and blood circulation. More generally, CED of radiopharmaceuticals in combination with radiosensitizers, such as ^18^F-FLT and 5FU, may provide a new effective treatment option for localized tumors and their metastases.

Moreover, considering the advances in TRT [41, 42], other radionuclides bound to molecules capable of reaching preferentially cancer cells could be injected directly into the primary tumor by CED, which is a clinical practice being increasingly applied to chemotherapy [20]. Recent developments in targeted PET imaging based on metabolism, angiogenesis, receptor-mediated antibodies, etc., may offer promising theranostic options, as already pointed out by others [43–45]. As in the present work, combining CED to the increased cancer cell specificity of these tracers relative to ^18^F-FDG could widen the range of PET radiopharmaceuticals potentially useful for therapy of not only well-localized tumors, but also their metastases.

## Conclusions

Collectively, the results obtained in this study indicate that *i.t.* administration of ^18^F-FLT may have definitive advantages for metabolic targeted radiotherapy (TRT). Compared to *i.t.* administration of ^18^F-FDG, tumor response to therapy can be slightly enhanced without detrimental consequences for most sensitive vital organs such as the brain, heart, and kidneys. While the radiation exposure of the bone marrow from ^18^F-FLT leaking from the tumor is a concern, it remains below the exposure from ^18^F-FDG. In either case, the *i.t.* administration concept for metabolic TRT of primary tumors has been established. Compared to external beam radiotherapy, metabolic TRT by *i.t.* infusion of ^18^F-FLT provides a sixfold gain in radiotherapeutic efficacy with less secondary effects to surrounding healthy tissues. Tumor response is further enhanced by the synergetic combination of ^18^F-FLT and the chemotherapeutic agent 5FU. Moreover, since *i.t.* ^18^F-FLT administration to a primary tumor provides significant uptake and residence time in distant tumors, it has the potential of either controlling or slowing the growth of metastases. Finally, PET imaging of positron-emitting radiolabeled compounds used for metabolic TRT allows direct visualization of the radiation source distribution and estimation of the local dose distribution during the entire course of treatment.

## Additional files

Additional file 1: **Figure S1.** The relationship of absorbed dose measured by Fricke dosimeter as a function of exposure activity of ^18^F-FDG. (TIF 11 kb)

Additional file 2: **Figure S2.** The relationship of cumulated activity detected by PET imaging per administered activity of ^18^F-FDG. (TIF 25 kb)

Additional file 3: Supplementary information. (DOCX 13 kb)

## Abbreviations

^18^F-FDG: 2-Deoxy-2-[^18^F]-fluoro-D-glucose
^18^F-FLT: 3′-Deoxy-3′-[^18^F]-fluorothymidine
5FU: 5-Fluorouracil
5Td: Five times tumor growth delay
CED: Convection-enhanced delivery
EBRT: External beam radiotherapy
EF: Enhancement factor
*i.p.*: Intraperitoneal
*i.t.*: Intratumoral
*i.v.*: Intravenous
LC/MS/MS: Liquid chromatography/tandem mass spectrometry
PET: Positron emission tomography
ROI: Region of interest
TAC: Time-activity curve
TGD: Tumor growth delay
TK1: Thymidine kinase 1
TRT: Targeted radiotherapy
