# Supplementary figures and images for: Intratumoral 18F-FLT infusion in metabolic targeted radiotherapy
Source: EJNMMI Res. 2019 Apr 11;9:33. doi: 10.1186/s13550-019-0496-7 (PMC6458198; doi:10.1186/s13550-019-0496-7)

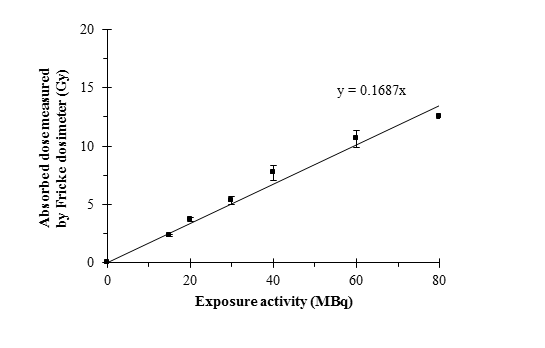

Supplement: Supplementary file 1 — Figure S1. The relationship of absorbed dose measured by Fricke dosimeter as a function of exposure activity of 18F-FDG. (TIF 11 kb) [file 13550_2019_496_MOESM1_ESM.tif]

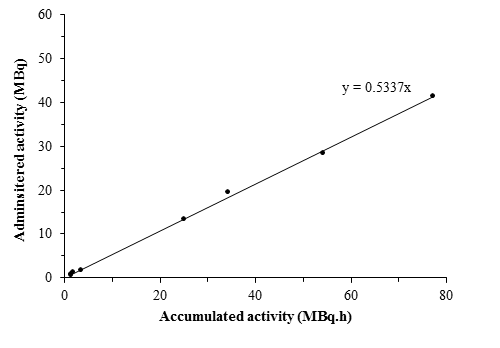

Supplement: Supplementary file 2 — Figure S2. The relationship of cumulated activity detected by PET imaging per administered activity of 18F-FDG. (TIF 25 kb) [file 13550_2019_496_MOESM2_ESM.tif]
