# Supplementary material for: Intratumoral 18F-FLT infusion in metabolic targeted radiotherapy
Source: EJNMMI Res. 2019 Apr 11;9:33. doi: 10.1186/s13550-019-0496-7 (PMC6458198; doi:10.1186/s13550-019-0496-7)
Supplement: Supplementary file 3 — Supplementary information. (DOCX 13 kb) [file 13550_2019_496_MOESM3_ESM.docx]

**Additional file 3**

**Article Title:**Intratumoral ^18^F-FLT infusion in metabolic targeted radiotherapy

Thititip Tippayamontri; Brigitte Guérin; René Ouellet; Otman Sarrhini; Jacques Rousseau; Roger Lecomte; Benoit Paquette; Léon Sanche

**DOI:**10.1186/s13550-019-0496-7
EJRE-D-18-00229.2

**Article: 496**

**Conversion factor for absorbed dose estimated by the Fricke chemical primary standard dosimeter**

The Fricke dosimeter is widely used as a chemical primary standard for dosimetry. Here, the transfer dosimeter is applied to a water phantom, under reference conditions. The relationship of absorbed dose per administered activity (MBq) is shown in the Figure S1. We summarize in this supplement, the method to transfer this standard to a mouse phantom. This is done by converting the signal in each pixel of a PET image to a corresponding absorbed dose of radiation in the scanned phantom. For instantaneous uptake with no biologic excretion, the total accumulated activity in a mouse phantom can be calculated as:

Ă (MBq.h) = 1.44 * A_0_ (MBq) * T_p_ (h) – (1)

Where Ă is the time-integrated activity (MBq.h)

A_0_ is the initial activity (MBq)

T_p_ is the physical decay half-life of the source (h)

Here, we can have a relationship of cumulated activity (MBq.h) and administered activity (MBq), as presented in the following Figure S2. Therefore, by taking into account Figure S1 and S2, we can derive a conversion factor as follows:

Fricke dosimeter: f(Gy) = aX + b = 0.1687X

PET imaging: f(1/MBq.h) = AX + B = 0.5337X

Conversion factor (C) = a*A = 0.1687*0.5337 = 0.09 (Gy/MBq.h)
